# Cardio-Respiratory and Muscle Oxygenation Responses to Submaximal and Maximal Exercise in Normobaric Hypoxia: Comparison between Children and Adults

**DOI:** 10.3390/biology12030457

**Published:** 2023-03-16

**Authors:** Anton Ušaj, Alexandros Sotiridis, Tadej Debevec

**Affiliations:** 1 Faculty of Sport, University of Ljubljana, SI-1000 Ljubljana, Slovenia; 2 School of Physical Education and Sport Science, National and Kapodistrian University of Athens, 115 27 Athens, Greece; 3 Department of Automatics, Biocybernetics and Robotics, Jozef Stefan Institute, SI-1000 Ljubljana, Slovenia

**Keywords:** hypoxemia, exercise, ventilation, muscle oxygenation, age, maturation

## Abstract

**Simple Summary:**

Exposure to altitude (hypoxia) is known to importantly challenge human physiology—particularly during physical exercise. While the effects of hypoxia on exercise performance have been extensively investigated in the last century, the effect of maturation remains poorly explored. In the present work, we aimed to compare the various physiological (cardiorespiratory and muscle oxygenation) responses to moderate- and high-intensity exercise in hypoxic condition in children as compared to adults to directly delineate the effects of aging. For this purpose, healthy prepubertal children and healthy adults underwent exercise testing in the laboratory under normal (normoxic) and simulated altitude (hypoxic) conditions with several physiological measures conducted continuously throughout both testing sessions. The comparison of the outcomes showed that hypoxia provoked similar exercise-related responses in most, but not all physiological responses in children and adults. From a practical point, these data suggest the comparable effects of hypoxia in children and adults with the observed differences warranting further investigation.

**Abstract:**

As differential physiological responses to hypoxic exercise between adults and children remain poorly understood, we aimed to comprehensively characterise cardiorespiratory and muscle oxygenation responses to submaximal and maximal exercise in normobaric hypoxia between the two groups. Following familiarisation, fifteen children (Age = 9 ± 1 years) and fifteen adults (Age = 22 ± 2 years) completed two graded cycling exercise sessions to exhaustion in a randomized and single-blind manner in normoxia (NOR; F_i_O_2_ = 20.9) and normobaric hypoxia (HYP; F_i_O_2_ = 13.0) exercises conditions. Age-specific workload increments were 25 W·3 min^−1^ for children and 40 W·3 min^−1^ for adults. Gas exchange and vastus lateralis oxygenation parameters were measured continuously via metabolic cart and near-infrared spectroscopy, respectively. Hypoxia provoked significant decreases in maximal power output P_MAX_ (children = 29%; adults 16% (F = 39.3; *p* < 0.01)) and power output at the gas exchange threshold (children = 10%; adults:18% (F = 8.08; *p* = 0.01)) in both groups. Comparable changes were noted in most respiratory and gas exchange parameters at similar power outputs between groups. Children, however, demonstrated, lower P_ET_CO_2_ throughout the test at similar power outputs and during the maintenance of $\dot{V}CO$_2_ at the maximal power output. These data indicate that, while most cardiorespiratory responses to acute hypoxic exercise are comparable between children and adults, there exist age-related differential responses in select respiratory and muscle oxygenation parameters.

## 1. Introduction

Exposure to altitude or hypoxia provokes many physiological responses, with their magnitude directly related to the hypoxic intensity and/or duration (i.e., hypoxic dose) [1,2,3,4]. Prominent acute hypoxia-related effects, aimed at counterbalancing the oxygen flux reductions, include increases in the resting alveolar rate and thereby the minute ventilation ($\dot{V}$_E_) and increased heart rate (HR) which, subsequently, is also reflected in the augmented cardiac output [3,5,6]. While hypoxia is known to reduce exercise capacity in all individuals [7,8], the effects of maturation/ageing on physiological responses during hypoxic exercise remain poorly investigated. Indeed, very few studies to date have investigated physiological responses to hypoxic exercise in children [9,10,11].

Several exercise-related physiological differences between children and adults are already prominent under normoxic conditions. Indeed, Dotan et al. [12] clearly showed differences in muscle motor units activation between children and adults. Both muscle glycogen concentration and phosphofructokinase activity are lower in children’s muscles [13,14]. There is also ample evidence to suggest various exercise-provoked cardiorespiratory and muscle blood flow dissimilarities, which might be directly related to growth and maturation [15,16]. 

Early work on hypoxic exercise-related differences between children and adults [17,18] did not elucidate any specific differences. Further, the data obtained by Kriemler and colleagues [10] during short term terrestrial altitude or our previous work in normobaric hypoxia [9] did not suggest any specific differential ventilatory responses in children. However, two important age-specific factors should be considered when assessing ventilatory responses to hypoxic exercise. The sensitivity of peripheral receptors for O_2_ seems to be higher (greater carotid bodies sensitivity), the and hypoxic end-tidal partial pressure of CO_2_ (P_ET_CO_2_) is lower in children [17]. In line with this, we recently observed age-specific cardiorespiratory and muscle oxygenation responses to hypoxic exercise at submaximal exercise intensities [11]. Particularly in adults, hypoxia seems to augment exercise $\dot{V}$_E_ at intensities above the gas exchange threshold (GET) and could maintain muscle blood oxygenation combined with an increased HR (e.g., augmented blood flow), whereas in children a greater increase in muscle blood perfusion, oxygen uptake and $\dot{V}$_E_ were observed at exercise intensities below the GET. It is of note that these differences were observed following nocturnal hypoxic residence and not upon acute hypoxic exposure, as employed in some previous work [17,18]. Additionally, recent work on subacute hypoxic exposures by Rieger and colleagues [19,20] demonstrated a higher incidence and severity of Acute Mountain Sickness but also showed comparable ventilatory responses and cerebrovascular responses in children as compared to adults. 

Given that the duration of the hypoxic exposure importantly modulates subsequent physiological responses, we hypothesized that some of the changes observed in $\dot{V}$_E_, HR as well as muscle blood perfusion and blood flow might not be observed upon acute hypoxic exercise. Due to the scarcity of relevant data and the contrasting results of the previous studies, we sought to further elucidate the physiological responses of children to exercise under acute hypoxic conditions, at both submaximal and maximal intensities. Altered muscle oxygenation patterns would indicate differential regulation of oxygen transport to the exercising muscles between children and adults. Furthermore, coupling (central) cardiorespiratory indices with (peripheral) muscle oxygenation parameters could provide some further insights into blood flow [21,22,23,24] and/or tissue perfusion [25] modulation during hypoxic exercise in children. Accordingly, we opted to comprehensively assess various cardiorespiratory and muscle oxygenation changes during acute hypoxic exercise in both adults and children at maximal and submaximal intensities as determined using the GET [26,27] and muscle oxygenation thresholds [28,29]. This would not only further our current understating of the basic age-related differences in acute hypoxia adaptation, but could also provide applied guidelines for families and groups with children for safe and enjoyable activities during high altitude sojourns. 

## 2. Materials and Methods

### 2.1. Participants and Ethical Approval

We recruited fifteen healthy, physically active children (7 girls and 8 boys; body mass = 33.1 ± 7.1 kg; stature = 1.40 ± 0.10 m; age = 9 ± 1 years. (mean ± SD)) and fifteen healthy, physically active adult males (body mass = 73.5 ± 5.6 kg; stature = 1.80 ± 0.05 m; age = 22 ± 2 years.) that completed all experimental sessions. All participants were low-altitude residents, did not take part in any type of hypoxic/altitude training before the study and were recreationally active ≥ four times·week^−1^ (endurance or resistance training activities). Exclusion criteria also comprised: prior (within two months preceding the experiments) exposure to terrestrial altitude (≥1700 m) and any chronic pulmonary, haematological and cardiac diseases. All participants (including children and their parents/legal guardians) were verbally informed regarding the aims of the study and the potential risks and challenges entailed in the experimental procedures and provided written informed consent before the study onset. Informed consent was also obtained from the children’s guardians. The study was approved by the National Committee for Medical Ethics at the Ministry of Health of the Republic of Slovenia (0120-101/2016-2) and conducted according to the current guidelines of the Declaration of Helsinki. 

### 2.2. Experimental Design and Protocol

During the preliminary session, participants performed a cycle ergometer testing and familiarised themselves with the laboratory settings and the envisaged exercise protocols. Subsequently, the participants completed two cycling graded exercise tests on two separate days in a randomized manner. Simple randomization (flipping a coin) was used to determine the first test-designated condition. One test was conducted under normoxic and one under normobaric hypoxic conditions. The testing protocol always commenced with two resting periods of five and two minutes, respectively, with the participants breathing ambient air in the former and the corresponding gas mixture (i.e., ambient air or hypoxic mixture) in the latter. The participants breathed the respective gas mixture via a face mask (Vmask, Hans Rudolph, Shawnee, KS, USA), attached directly to a metabolic cart flowmeter (Quark CPET, Cosmed, Rome, Italy) and a two-way low resistance valve (2700 NRBV; Hans Rudolph Inc., Shawnee, KS, USA) throughout all tests. We used specific children and adult mask sizes to ensure optimal fitting and reduced the mask dead space in children and adults, respectively. During the normoxic trials, the valve was connected to a 200-L Douglas bag containing ambient air (NOR; F_i_O_2_ = 0.21; P_i_O_2_ = 19.7 kPa), and during the hypoxic trials, the Douglas bag contained a normobaric hypoxic gas mixture (HYP; F_i_O_2_ = 0.13; P_i_O_2_ = 12 kPa). The resting period was followed by a three-minute warm-up at 40 W for the adults and 25 W for the children. Subsequently, the workload was increased by 40- or 25-W step increments every three minutes for adults and children, respectively. The participants were required to maintain a pedalling cadence of 60–70 rpm throughout the test. As the exercise intensity/power output increased, the test was terminated when the participants were unable to sustain the prescribed pedalling cadence despite strong verbal encouragement. This intensity was denoted as maximal (MAX).

### 2.3. Measurements

#### 2.3.1. Baseline Anthropometric Assessment

Baseline body mass and stature measurements were performed using a stadiometer scale (Libela ELSI, Celje, Slovenia). The whole-body fat percentage was estimated using the Jackson and Pollock equation [30] from nine skin-fold measurement sites [triceps, subscapular, chest, suprailiac, abdominal, thigh (3 sites) and inguinal]. 

#### 2.3.2. Gas Exchange

Continuous breath-by-breath gas exchange and ventilatory parameters were recorded throughout the testing protocol using a metabolic cart (Quark CPET, Cosmed, Rome, Italy). Before each test, the O_2_ and CO_2_ sensors of the metabolic cart were calibrated using two calibration gas mixtures (CO_2_ fraction: 5% and O_2_ fraction: 16%) along with the flowmeter calibration in line with the manufacturer’s instructions. HR and SpO_2_ were measured continuously using a fingertip pulse oximetry device 3100 WristOx (Nonin Medicals, Plymouth, MN, USA). Attainment of the maximum oxygen uptake ($\dot{V}O$_2MAX_), defined as the highest oxygen consumption $\dot{V}O$_2_ values across 30 s, was based on at least one of the following established criteria [31]: (i) cycling cadence < 60 rpm, (ii) plateau in $\dot{V}O$_2_ and/or (iii) respiratory exchange ratio (RER) ≥1.1. The reported cardiorespiratory and end-tidal gas pressure values at the GET and MAX were also calculated as 30-s averages just before the GET and during the last phase of the testing, respectively.

#### 2.3.3. Muscle Oxygenation

Near-infrared spectroscopy (NIRS; Oxymon MK III, Artinis Medical systems, Zatten, Netherlands) was employed to assess the relative changes in oxygenated haemoglobin (O_2_Hb) and deoxygenated haemoglobin (HHb) concentrations. Total haemoglobin (tHb), an index of tissue perfusion (muscle blood volume), was calculated from the O_2_Hb and HHb values. The NIRS probes were positioned over the (distal) belly of the right vastus lateralis muscle. This measurement site represents one of the most used locations to assess the lower extremities’ muscle oxygenation during cycling-type exercise [32,33,34] and, thus, also enables for comparisons with other similar studies. Ink marks applied to the skin ensured that the probes were positioned at the same site in both the NOR and HYP tests. The median fat layer above the measurement location, assessed using a calibrated skinfold calliper, was comparable between the two cohorts (adults = 14 ± 6 mm; children = 16 ± 3 mm; *p* = 0.12).

The basic principles and theory of NIRS as well as their application to the muscle oxygenation assessment are extensively detailed elsewhere [32,33,35]. Briefly, the laser light of 760 nm is emitted through the skin and absorbed by the haemoglobin. The magnitude of the absorption of near infra-red light is dependent on the oxygen bound to the haemoglobin (Hb), specifically if Hb is saturated with O_2_ (oxyhemoglobin; O_2_Hb) or not (deoxyhemoglobin; HHb). A portion of the light is then reflected to the optical detector. The signal is subsequently analysed to obtain values of O_2_Hb, HHb and the total haemoglobin content (tHb). The data were continuously recorded and calculated at a 50-Hz frequency. Although it is assumed that alterations in O_2_Hb and HHb reflect changes in muscle $\dot{V}O$_2_, any concomitant alterations in muscle blood flow and perfusion must also be considered. Acceptable reproducibility of the muscle deoxygenation measurement using the NIRS technique in children has previously been demonstrated [36]. It is also worth mentioning that the continuous wave NIRS technique is very sensitive to the thickness of the subcutaneous fat layer, which may elicit errors in the results of muscle oxygenation [37,38]. It is also important to note that relative rather than absolute values of all three parameters were used. Thus, we report the relative changes in arbitrary units (AU) from the baseline, resting average values of each respective parameter, thus also ensuring that the data across participants are comparable. Accordingly, for example, the exercise-related reductions in O_2_Hb were reflected in the negative AU values. 

### 2.4. Data Analysis and Processing

All statistical analyses were performed using the SPSS package (version 22.0, SPSS Inc., Chicago, IL, USA) and Sigma Plot 11 (Systat, San Hose, CA, USA). Data are presented as means ± standard deviations throughout the manuscript. The data were initially tested for normality of distribution using the criteria of skewness or kurtosis. Significant differences across environmental conditions and exercise intensity were performed using a paired t-test and ANOVA for repeated measurements for adults and children separately for $\dot{V}$_E_, $\dot{V}CO$_2_, $\dot{V}O$_2_, P_ET_O_2_, P_ET_CO_2_, respiratory frequency (f_R_), RER, HR, O_2_Hb, HHb and tHb. Two-way ANOVA was used to compare the threshold and maximal intensity parameters between NOR and HYP conditions and between adults and children. When a main effect was noted, a post hoc test (Bonferroni) was employed to determine the specific differences. The study sample size (15 participants per group) was based on our previous similar investigations [11,34], with a posteriori analysis indicating that a statistical power >0.80 was achieved for all main cardiorespiratory parameters comparisons. As mentioned previously, the only exception were the muscle oxygenation data, where the data was only analysed within groups.

During the exercise protocol, minute ventilation ($\dot{V}$_E_), CO_2_ output ($\dot{V}CO$_2_) and all three parameters of muscle oxygenation (O_2_Hb, HHb and tHb) demonstrated a two-phase response; a response characterized by the intersection point of the two best $\dot{V}CO$_2_ fitted regression lines in the low and steep parts of the $\dot{V}CO$_2_ vs. $\dot{V}O$_2_ diagrams. The intersection point was located using Matlab R2019b (Mathworks Inc., Natick, MA, USA) and was further used to detect the gas exchange threshold (GET) [11,26]. As O_2_Hb, HHb and tHb were previously shown to follow similar patterns [28,29,35], the thresholds were also determined from the three NIRS-derived values (O_2_Hb_Th_, HHb_Th_ and tHb_Th_, respectively). As extensively detailed previously (See Figure 1 in [11]), the respective NIRS parameters thresholds were also determined using the intersection of the best fitting regression lines of the two-phase responses of the respective parameters using Matlab R2019b. In addition to the power output (P) of the GET (P_GET_), the following relative parameters were also calculated: $\dot{V}$_E_ at the GET ($\dot{V}$_E GET_), $\dot{V}O$_2_ at the GET ($\dot{V}O$_2 GET_), HR at the GET (HR_GET_), $\dot{V}CO$_2_ at the GET ($\dot{V}CO$_2 GET_), O_2_Hb at O_2_HbT (O_2_Hb_O2HbTh_), HHb at HHbT (HHb_HHbTh_) and tHb at tHbT (tHb_tHbTh_). 

The effects of hypoxia were determined by comparing the differences observed between the NOR and HYP conditions at two distinct exercise intensities: a submaximal intensity, corresponding to the GET in each environmental condition, and a maximal intensity (MAX). Comparisons with values normalized to body mass were conducted when appropriate.

## 3. Results

### 3.1. Submaximal and Maximal Cardiorespiratory Responses

**Figure 1:**
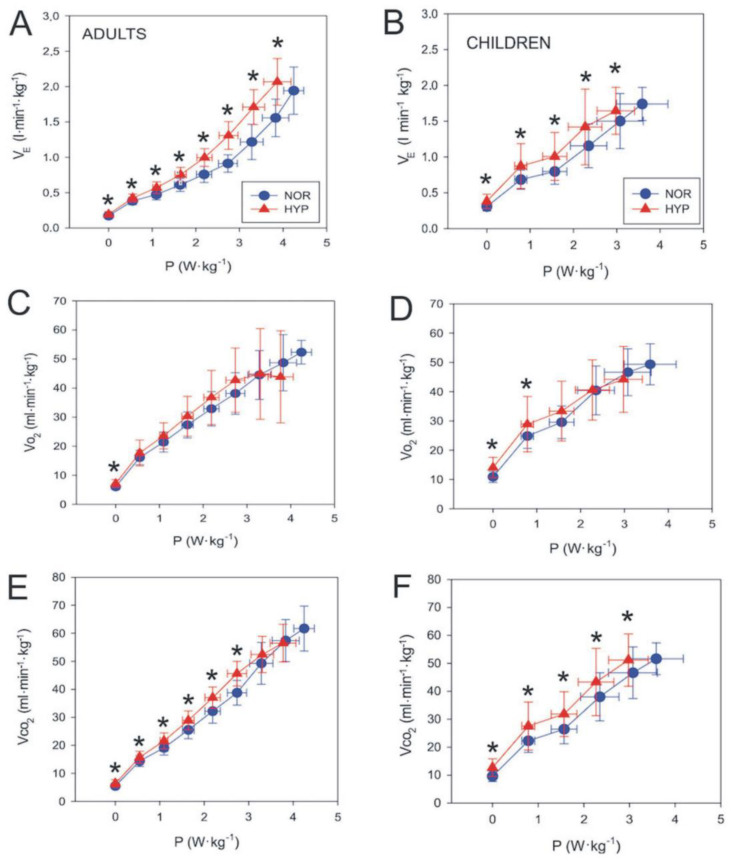
Resting and exercising values of minute ventilation ($\dot{V}$_E_; panels (**A**,**B**)), oxygen consumption $\dot{V}O$_2_; panels (**C**,**D**)) and carbon dioxide output ($\dot{V}CO$_2_; panels (**E**,**F**)) for both adults and children. Values are reported as means (SD). Significant post hoc differences: * (*p* < 0.05) denotes significantly different values compared to NOR.

**Figure 2:**
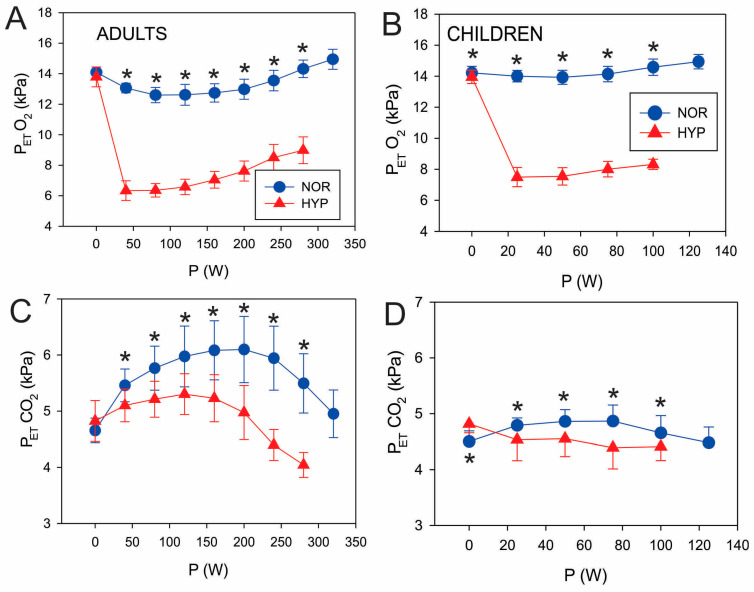
Resting and exercising values of end-tidal oxygen pressure (P_ET_O_2;_ panels (**A**,**B**)) and end-tidal carbon dioxide pressure (P_ET_CO_2_; panels (**C**,**D**)) for both adults and children. Values are reported as means (SD). Significant post hoc differences: * (*p* < 0.05) denotes significantly different values compared to NOR.

Relative $\dot{V}$_E_ values increased throughout the graded test to a greater extent in HYP as compared to NOR conditions in both adults and children (Figure 1A,B). If the lower exercise intensities (40 W in adults as well as 25 and 50 W in children where $\dot{V}O$_2_ values were higher in HYP) are excluded, $\dot{V}O$_2_ increased similarly in the NOR and HYP conditions in both adults and children (Figure 1C,D). In contrast, the $\dot{V}CO$_2_ values increased more in HYP than in NOR in both groups (Figure 1E,F). Furthermore, P_ET_O_2_ and P_ET_CO_2_ decreased in HYP vs. NOR conditions both in adults and children (Figure 2). While the maximal difference between P_ET_CO_2_ in NOR and HYP during the graded test exceeded 1 kPa (Figure 2C) in adults, the respective decrease in children did not exceed 0.5 kPa (Figure 2D). HR was higher during the HYP than during the NOR exposure throughout the graded test. 

As depicted in Table 1, P_GET_ decreased by about 18% in adults and by about 10% in children under hypoxic conditions (F = 8.08; *p* = 0.01), with the values remaining higher in adults than children (F = 72.88; *p* < 0.01). No interaction effect between groups (*p* = 0.14) was detected. While HYP did not impact the GET-corresponding metabolic parameters $\dot{V}O$_2_, $\dot{V}CO$_2_ and $\dot{V}$_E_, the differences in those parameters between adults and children remained significant (Table 1). The same goes for the f_R_ and RER with the former significantly higher and the latter significantly lower in children compared to adults. In contrast, P_ET_O_2 GET_ as well as P_ET_CO_2GET_ decreased similarly in HYP (Table 1). HR_GET_ was not significantly influenced by HYP conditions, and despite a tendency for differences between children and adults, it was comparable between groups (Table 1).

As noted in Table 2, P_MAX_ decreased in HYP conditions by about 16% (*p* < 0.01) in adults and 29% (*p* < 0.01) in children, with the reduction being significantly greater in children (F = 39.30; *p* < 0.01) and no interaction between groups (*p* = 0.20). Children displayed about 9% lower $\dot{V}$_E MAX_ values during HYP conditions as compared to adults with 2% (F = 4.33; *p* = 0.04). However, this HYP-related decrease was not significant (F = 1.01; *p* = 0.32; Table 2). Therefore, this pattern of non-significant changes mainly matched the pattern of $\dot{V}$_E GET_ changes. Normalized $\dot{V}O$_2 MAX_ decreased by 10% in adults and by 12% in children during exercise in HYP conditions (F = 9.15; *p* < 0.01; Table 2) with, again, no interaction between groups (*p* = 0.77). Normalized $\dot{V}CO$_2 MAX_ values decreased during HYP exposure by about 20% (*p* < 0.01) in adults but remained similar (*p* = 0.07) despite a tendency for a decrease (9%) in children, with a tendency for significant differences between children and adults (F = 4.17; *p* = 0.05; Table 2). No significant differences were observed between conditions nor groups in both; HR_MAX_ reached similar values during HYP conditions as well as NOR conditions across groups (Table 2) and matched the pattern of HR_GET_ changes. Absolute P_ET_O_2_ values were lower in HYP (F = 1895; *p* = 0.00; Table 2) conditions. It was larger in adults (F = 5.41; *p* = 0.04), with no significant interaction between groups. P_ET_CO_2 MAX_ decrease due to hypoxia (F = 28.96; *p* < 0.01) is larger in adults than in children (F = 5.08; *p* = 0.03; Table 2), with no interaction between groups (*p* = 0.81; Table 2). However, the general pattern of P_ET_CO_2_ changes (decrease) matched the changes in P_ET_CO_2 GET_. SpO_2_ decreased similarly by about 20% in HYP conditions for adults and children.

### 3.2. Submaximal and Maximal Muscle Oxygenation Responses

**Figure 3:**
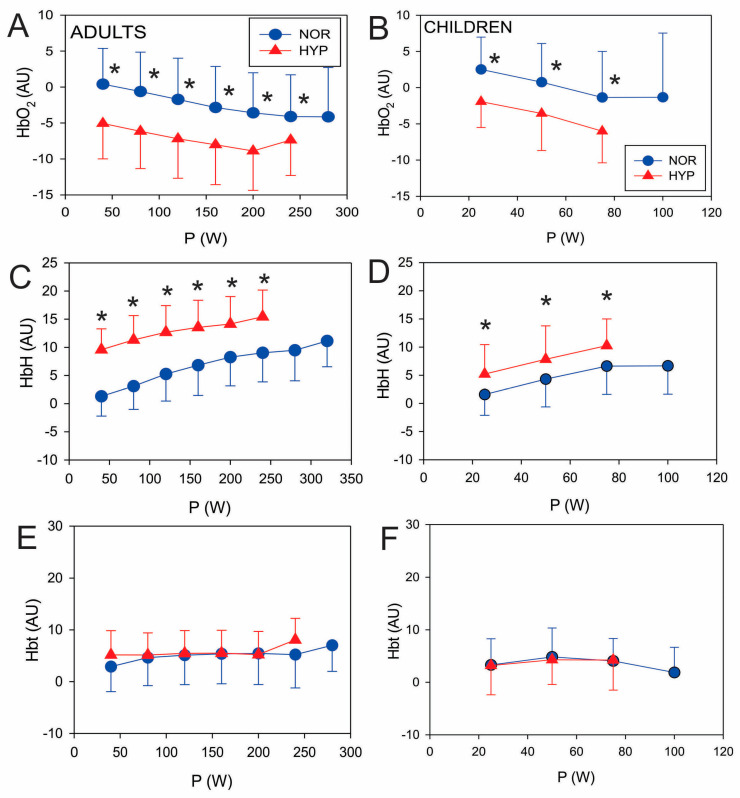
Exercising values of oxygenated haemoglobin (O_2_Hb; panels (**A**,**B**)), deoxygenated haemoglobin (HHb; panels (**C**,**D**)) and total haemoglobin (tHb; panels (**E**,**F**)) for both adults and children. Values are reported as means (SD). Significant post hoc differences: * (*p* < 0.05) denotes significantly different values compared to NOR.

Generally, both adults and children displayed similar muscle oxygenation responses during the exercise testing (Figure 3). O_2_Hb decreased in a parallel manner in the NOR and HYP conditions with about a 5–7 AU difference in adults (Figure 3A) and less than 5 AU in children (Figure 3B). HHb also increased similarly. Adults started with a difference between the NOR and HYP conditions of about 9 AU at 40 W (Figure 3C) and reached about 5 AU at 250 W (Figure 3C). Similarly, children’s HHb also increased parallel between the NOR and HYP values (Figure 3D) yet with a smaller difference (less than 5 AU) in relation to adults. tHb showed only a small increase from its resting level in both adults and children, and was not influenced by HYP conditions (Figure 3E,F).

Both muscle oxygenation thresholds, O_2_Hb_Th_ and HHb_Th_, were lower in HYP conditions in adults by about 16% (*p* = 0.04) and 21% (*p* < 0.01), respectively (Table 3). In children, O_2_Hb_Th_ decreased for about 24% (*p* < 0.01) and HHb_Th_ for about 35% (*p* < 0.01) (Table 3). Significant changes occurred in adults and children when muscle oxygenation thresholds were represented by the corresponding O_2_Hb and HHb but not the tHb values. Indeed, the observed changes in O_2_Hb and HHb in HYP conditions (Table 3) in adults and children seem to indicate distinct patterns in the muscles as opposed to cardiorespiratory parameters at the GET (Table 1). 

## 4. Discussion

This study aimed to further explore the potential differences between children and adults in cardiorespiratory and muscle oxygenation responses to hypoxic exercise. We tackled this by analysing and comparing all the cardiorespiratory and muscle oxygenation data from the graded exercise tests at submaximal and maximal intensities across groups. Overall, our results show that P_GET_ and P_MAX_ significantly decreased in an hypoxic environment in both children and adults. These patterns were generally matched by changes in P_ET_CO_2_ corresponding to both the GET and MAX, while some parameters such as $\dot{V}$_E GET_ and $\dot{V}$_E MAX_, HR_GET_, HR_MAX_, HbO_2GET_, HHb_GET_ and tHb_GET_ seem similar between groups during hypoxic exercise. On the other hand, other parameters such as P_ET_CO_2_ throughout the graded test, as well as $\dot{V}CO$_2_ at the GET and MAX intensities, show a distinct response between adults and children. These differences cannot be explained solely based on different body mass characteristics, as a very limited or negligible effect of the body mass normalization procedure was observed in the present work. 

During graded exercise testing, $\dot{V}$_E_ was significantly higher in hypoxic than normoxic conditions in both groups. The difference between the two environmental conditions became clearer during moderate-to-high intensities—at intensities exceeding the GET. However, $\dot{V}$_E_ HYP values followed $\dot{V}$_E_ NOR values without the deviation from normoxic values previously noted in adults (Figure 1A,B, [11]). The additional activation of Type IIa instead of predominantly active Type I muscle fibres which were activated at intensities below the GET might be a potential explanation for the exponential rise in $\dot{V}$_E_ observed in adults and in particular explains the absence of this phenomenon in children [12]. One possible explanation for the lack of $\dot{V}$_E_ changes could also be related to the fact that children possess a lower percentage of Type IIa muscle fibres [12,39], which might play an important role in modulating higher intensity exercise responses (e.g., above the GET). Interestingly, we also did not observe any specific effect of hypoxia on the respiratory frequency in either group. While the maximal exercise values were comparable between the groups, and in line with those reported in the literature [40,41], children exhibited significantly higher absolute respiratory frequencies at the GET (i.e., submaximal intensities) compared to adults, which is probably mostly related to the differences in body sizes and metabolic demands as these data cannot reasonably be normalised to BM.

The above-noted respiratory differences between children and adults could also have influenced the observed differences in P_ET_O_2_ and P_ET_CO_2_. The lower P_ET_CO_2_ during hypoxic exposure in children may be the effect of higher $\dot{V}$_E_ under hypoxia. This observation was the first differential hypoxia-related characteristic observed between children and adults in the present study. Even though we have observed a similar phenomenon recently [11], whether this is a result of the higher sensitivity of children to hypoxia remains unclear despite the evidence by Springer and colleagues [17,18,42], which clearly indicated that peripheral chemoreceptors exert a significantly greater influence on hypoxic exercise-related hyperpnea in children compared to adults. Given the paramount importance of peripheral afferent inputs (i.e., carotid bodies) on ventilatory control [43], the peripheral chemoreceptors’ maturation (sensitivity and subsequent contribution to ventilatory changes) with aging requires further scrutiny. In addition to the ventilatory underpinnings, the reported differences in substrate utilization during exercise between children and adults [44,45] could also have played a role. Indeed, ample evidence suggests that children are generally more reliant on fat and less on carbohydrate energy utilisation, particularly during submaximal exercise, which then reflects in a lower RER at the same absolute intensities [44]. The present data further reinforce this notion and indicate that the same holds true for hypoxic exercise at submaximal and not maximal intensities. Regarding the hypoxia-related decrease in P_GET_ and the power outputs at submaximal thresholds, one could also speculate that this is a part of a hypoxia-related homeostatic regulation that aims to minimize the energy cost of the hypoxic response and regulate appropriate energy availability. At the muscle level, the optimization of oxygen flux to mitochondria is achieved by the precise regulation of blood perfusion distribution rather than a tachycardia-mediated increase in blood flow [24,25,46,47]. Using the employed methodology, we were unfortunately not able to accurately assess whether this was more efficient in adults than in children. Normalization to BM at submaximal intensities did not reduce differences between adults and children except in $\dot{V}O$_2 GET_ and HR_GET_, where differences between adults and children became negligible. Even normalization to BM^0.67^ [48] did not provide any further insights (analysis results not presented in the manuscript). Therefore, other factors, rather than BM independently, probably underline the differential hypoxic responses between adults and children. 

During maximal exercise, the hypoxia-induced reduction in the maximal power output (P_MAX HYP_) was about 16% in adults and 29% in children—in line with the pattern of decreased P_GET_. While a decrease of 16% can be explained by a comparable 10% decrease $\dot{V}O$_2 MAX_ in adults, the decrease of 29% observed in children cannot be solely attributed to the 12% decrease in children’s $\dot{V}O$_2 MAX_ [5]. Potential differences in motivation and the sensation of fatigue could have played roles. It is of note that Kreimler et al. [10] found a similar decrease in the peak power output of 19% and a concomitant 20% decrease in $\dot{V}O$_2 MAX_ in children during exposure to a terrestrial altitude of 3400 m. In contrast to P_MAX_, the $\dot{V}$_E MAX_ did not decrease during HYP exposure in neither adults nor children. On the other hand, the $\dot{V}CO$_2 MAX HYP_ decreased by 20% in adults but remained relatively similar (despite a 9% decrease in children). This was the second characteristic which significantly differentiated the response of children and adults to hypoxia during exercise. Our values were slightly different but comparable to those reported previously by Kriemler et al. [10]. Interestingly, we also did not observe any differences between children and adults in submaximal and maximal HR responses to hypoxic exercise. Given that changes in HR, and therefore cardiac output, are among the most prominent acute adaptations to hypoxia, this finding suggests that exercise-related cardiac adaptation seems comparable between the two age groups under the prevailing conditions of the present study.

No specific differences in the measured muscle oxygenation parameters were noted during submaximal exercise intensities between children and adults, and the results of all parameters are somewhat expected to be uniform in both cohorts (i.e., a relative decrease in O_2_Hb and an increase in HHb under hypoxic vs. normoxic conditions). It is, however, of note that at maximal power outputs, the children’s muscle HHb response to hypoxia was somewhat different to that of adults. This finding is somewhat contrasting to our previous work [11] where children exhibited a significantly higher tHb concentration during hypoxic exercise at submaximal exercise intensities. As our experimental design does not allow for us to directly elucidate the exact reasons for these discrepant muscle oxygenation outcomes, they are probably related to different hypoxic exposure times before the testing. 

### Methodological Considerations

Even though the present study provides further insight into hypoxic exercise-associated physiological responses in relation to aging, there are a few limitations we need to acknowledge. Differences between hypoxic doses applied in our recent study [11] and the present study may contribute to the discrepancies observed between adults and children to hypoxic exposure. Namely, the hypoxic dose in our previous study was defined by the difference between the NOR conditions (about 1000 m of terrestrial altitude where altitude training was performed) and HYP conditions (simulated 3000 m), therefore about 2000 m and about 12 h of exposure throughout the night and following morning, preceding the second testing period in HYP conditions. The hypoxic dose of the present study was about 3500 m of simulated altitude (from 300 m of terrestrial altitude to 3500 m of simulated altitude) and without any “acclimatization” before testing (acute hypoxic exposure). Despite being more intense, the shorter hypoxic dose in the present study elicited similar cardiorespiratory responses but larger differences in muscle oxygenation in adults when compared with results of our previous study [11]. It could be speculated that the intensity of the hypoxic dose importantly modulates the physiological characteristic related to the observed exercise responses. Nevertheless, the importance of the duration of hypoxic exposure remains to be further elucidated. Additionally, whether children’s tHb might have increased because of prolonged (nocturnal) and low-intensity hypoxic dose as opposed to a shorter but more intense hypoxic dose also remains unclear. Regarding the age-related differences in muscle oxygenation and, interrelated, the respiratory and cardiac responses to hypoxic exercise, future work should also implement an assessment of respiratory muscle oxygenation (e.g., intercostal muscles) [49]. Furthermore, the characterisation of cardiorespiratory coherence, known to be importantly modulated by hypoxia during rest [50] and exercise [51], would also seem warranted to gain further insights into the age-related divergencies in cardiac and respiration neural regulation [52]. Finally, we want to emphasize that the present data were obtained using simulated (normobaric) hypoxic exposures, and the limitations pertinent to this aspect as compared to real (hypobaric hypoxic) altitude exposure need to be considered [53].

## 5. Conclusions

Overall, our results indicate that hypoxia-related exercise responses in children and adults are comparable in most of the measured ventilatory and oxygenation parameters (P_GET_, P_MAX_, $\dot{V}$_E GET_, HbO_2GET_, HHb_GET_ and tHb_GET_) but are clearly different in some (P_ET_CO_2_ and $\dot{V}CO$_2 MAX HYP_). Importantly, the observed dissimilarities do not seem to be directly related to morphological differences, as only a small influence of body mass normalization was noted. The detected differences in these physiological parameters were small and fragmental, particularly in contrast to clear and robust decreases in submaximal and maximal power outputs observed in both tested groups. While this study provides further insights into hypoxia-related cardiorespiratory and muscle oxygenation modulation differences between children and adults, further—especially longer-term adaptation—studies seem warranted.

## Figures and Tables

**Table 1:** Cardiorespiratory and gas exchange values in adults and children at submaximal intensities during normoxic (NOR) and hypoxic (HYP) exercise testing sessions.

	Adults		Children		*p* Value	*p* Value
	NOR	HYP	NOR	HYP	(NOR:HYP)	(Adults:Children)
P_GET_ (W·kg^−1^)	2.88 ± 0.50	2.37 ± 0.48	1.43 ± 0.44	1.29 ± 0.31	0.01	<0.01
$\dot{V}$_E GET_ (L·min^−1^·kg^−1^)	0.90 ± 0.17	0.94 ± 0.17	0.74 ± 0.11	0.87 ± 0.27	0.09	0.03
$\dot{V}O$_2 GET_ (mL·min^−1^·kg^−1^)	39 ± 5	35 ± 3	34 ± 5	33 ± 9	0.19	0.05
$\dot{V}Oo$_2 GET_ (mL·min^−1^·kg^−1^)	36 ± 7	35 ± 7	24 ± 4	28 ± 7	0.58	<0.01
f_R GET_ (breaths·min^−1^)	25 ± 5	26 ± 6	36 ± 8	40 ± 12	0.07	<0.01
RER _GET_	0.97 ± 0.06	0.98 ± 0.14	0.88 ± 0.04	0.94 ± 0.06	0.08	<0.01
HR_GET_ (min^−1^)	161 ± 24	158 ± 14	147 ± 13	157 ± 11	0.31	0.06
P_ET_O_2GET_ (kPa)	12.8 ± 0.7	7.0 ± 0.7	13.8 ± 0.4	7.0 ± 1.4	<0.01	0.01
P_ET_CO_2GET_ (kPa)	6.1 ± 0.6	5.2 ± 0.4	4.9 ± 0.2	4.6 ± 0.3	<0.01	<0.01

Values are mean ± SD. P, power output; GET, gas exchange threshold; $\dot{V}$_E_, minute ventilation; $\dot{V}O$_2_, oxygen uptake; $\dot{V}CO$_2_, carbon dioxide output; f_R_, respiratory frequency; RER, respiratory exchange ratio; HR, heart rate; P_ET_O_2_, end-tidal O_2_; P_ET_CO_2_, end-tidal CO_2_.

**Table 2:** Cardiorespiratory and gas exchange values in adults and children at maximal intensities during normoxic (NOR) and hypoxic (HYP) exercise testing sessions.

	Adults		Children		*p* Value	*p* Value
	NOR	HYP	NOR	HYP	(NOR:HYP)	(Adults:Children)
P_MAX_ (W·kg^−1^)	4.52 ± 0.42	3.80 ± 0.43	3.80 ± 0.73	2.70 ± 0.56	<0.01	<0.01
$\dot{V}$_E MAX_ (L·min^−1^·kg^−1^)	1.95 ± 0.30	1.92 ± 0.29	1.81 ± 0.43	1.64 ± 0.46	0.32	0.04
$\dot{V}O$_2 MAX_ (mL·min^−1^·kg^−1^)	53 ± 5	48 ± 4	50 ± 7	44 ± 10	<0.01	0.10
$\dot{V}CO$_2 MAX_ (mL·min^−1^·kg^−1^)	64 ± 6	51 ± 15	54 ± 11	49 ± 11	<0.01	0.05
f_R MAX_ (breaths·min^−1^)	52 ± 6	52 ± 8	59 ± 10	54 ± 14	0.28	0.25
RER	1.22 ± 0.06	1.15 ± 0.22	1.07 ± 0.06	1.15 ± 0.20	0.76	0.06
HR_MAX_ (L·min^−1^)	182 ± 11	184 ± 8	186 ± 12	180 ± 10	0.72	0.98
SpO_2 MAX_ (%)	94 ± 2	75 ± 5	96 ± 1	77 ± 4	<0.01	0.05
P_ET_O_2 MAX_ (kPa)	15.0 ± 0.4	8.8 ± 0.8	12.4 ± 0.3	8.3 ± 0.5	<0.01	0.02
P_ET_CO_2 MAX_ (kPa)	4.9 ± 0.4	4.2 ± 0.3	4.5 ± 0.2	4.3 ± 0.3	<0.01	0.03

Values are mean ± SD. P, power output; $\dot{V}$_E_, minute ventilation; $\dot{V}O$_2_, oxygen uptake; $\dot{V}CO$_2_, carbon dioxide output; f_R_, respiratory frequency; RER, respiratory exchange ratio; HR, heart rate; SpO_2_, capillary oxyhemoglobin saturation; P_ET_O_2_, end-tidal O_2_; P_ET_CO_2_, end-tidal CO_2_.

**Table 3:** Power outputs at muscle oxygenation thresholds and muscle oxygenation (vastus lateralis) parameters at respective thresholds in adults and children at submaximal intensities during normoxic (NOR) and hypoxic (HYP) exercise testing sessions.

	Adults			Children		
	NOR	HYP	*p* Value	NOR	HYP	*p* Value
O_2_Hb_Th_ (W)	199 ± 61	167 ± 38	0.04	54 ± 17	41 ± 13	0.01
HHb_Th_ (W)	215 ± 40	166 ± 33	<0.01	58 ± 24	38 ± 14	0.01
tHb_Th_ (W)	183 ± 73	174 ± 42	0.70	57 ± 19	48 ± 18	0.10
O_2_Hb_O2HbTH_ (AU)	−2.4 ± 4.6	−7.7 ± 5.2	0.01	2.1 ± 3.3	−1.2 ± 2.7	0.01
HHb_HHbTh_ (AU)	9.2 ± 5.4	12.9 ± 5.3	0.01	1.0 ± 3.3	4.5 ± 4.8	0.02
tHb_tHbTh_ (AU)	5.1 ± 5.1	5.9 ± 4.4	0.60	2.6 ± 4.7	3.2 ± 5.9	0.88

Values are mean ± SD. O_2_Hb, oxygenated haemoglobin; TH, threshold; HHb, deoxygenated haemoglobin; tHb, total haemoglobin: W, watt; AU, arbitrary units.

## Data Availability

Due to ethical and privacy issues, the full data set is not publicly available but can be obtained from the corresponding author upon reasonable request.
